# Sweat rate and sweat composition following active or passive heat re-acclimation: A pilot study

**DOI:** 10.1080/23328940.2020.1826287

**Published:** 2020-10-11

**Authors:** Lisa Klous, Cornelis de Ruiter, Puck Alkemade, Hein Daanen, Nicola Gerrett

**Affiliations:** Department of Human Movement Sciences, Faculty of Behavioural and Movement Sciences, Vrije Universiteit Amsterdam, Amsterdam Movement Sciences, Amsterdam, The Netherlands

**Keywords:** Controlled hyperthermia, heat acclimation, hot water immersion, local sweat rate, sweat composition

## Abstract

The purpose of this study was to investigate local sweat rate (LSR) and sweat composition before and after active or passive heat re-acclimation (HRA). Fifteen participants completed four standardized heat stress tests (HST): before and after ten days of controlled hyperthermia (CH) heat acclimation (HA), and before and after five days of HRA. Each HST consisted of 35 min of cycling at 1.5W·kg^−1^ body mass (33°C and 65% relative humidity), followed by a graded exercise test. For HRA, participants were re-exposed to either CH (CH-CH, *n* = 6), hot water immersion (water temperature ~40°C for 40 min; CH-HWI, *n* = 5) or control (CH-CON, *n* = 4). LSR, sweat sodium, chloride, lactate and potassium concentrations were determined on the arm and back. LSR increased following HA (arm +18%; back +41%, *P ≤ * 0.03) and HRA (CH-CH: arm +31%; back +45%; CH-HWI: arm +65%; back +49%; CH-CON arm +11%; back +11%, *P ≤ *0.021). Sweat sodium, chloride and lactate decreased following HA (arm 25–34; back 21–27%, *P* < 0.001) and HRA (CH-CH: arm 26–54%; back 20–43%; CH-HWI: arm 9–49%; back 13–29%; CH-CON: arm 1–3%, back 2–5%, *P* < 0.001). LSR increases on both skin sites were larger in CH-CH and CH-HWI than CH-CON (*P* ≤ 0.010), but CH-CH and CH-HWI were not different (*P* ≥ 0.148). Sweat sodium and chloride conservation was larger in CH-CH than CH-HWI and CH-CON on the arm and back, whilst CH-HWI and CH-CON were not different (*P* ≥ 0.265). These results suggest that active HRA leads to similar increases in LSR, but more conservation of sweat sodium and chloride than passive HRA.

**Abbreviations:** ANOVA: Analysis of variance; ATP: Adenosine triphosphate; BSA (m^2^): Body surface area; CH: Controlled hyperthermia; CH-CH: Heat re-acclimation by controlled hyperthermia; CH-CON: Control group (no heat re-acclimation); CH-HWI: Heat re-acclimation by hot water immersion; CV (%): Coefficient of variation; dt (min): Duration of a stimulus; F: Female; GEE: Generalized estimating equations; HA: Heat acclimation; HRA : Heat re-acclimation; HST: Heat stress test; LSR (mg·cm^−2^·min^−1^) : Local sweat rate; LOD (mmol·L^−1^): Limit of detection; M: Male; $m_{x}$ (mg): Mass of x; RH (%): Relative humidity; RT: Recreationally trained; SA (cm^2^): Surface area; t (min): Time; T: Trained; T_sk_ (°C): Skin temperature; T_re_ (°C): Rectal temperature; USG : Urine specific gravity; VO_2peak_ (mL·kg^−1^·min^−1^): Peak oxygen uptake; WBSL (L): Whole-body sweat loss; WBSR (L·h^−1^): Whole-body sweat rate

## Introduction

Sweat contains numerous important ions for maintaining body fluid balance, epidermal barrier homeostasis and antimicrobial function of the skin [1–4]. In a hot environment or during exercise, sweat production is increased to facilitate heat loss. Sweating is an important heat loss mechanism, yet a disadvantage of an elevated sweat production is a concomitant elevation in ion losses, potentially disturbing fluid balance. An important eccrine sweat gland adaptation from heat acclimation (HA) is the improved conservation of sodium and chloride in the presence of an increased local sweat rate (LSR) [5–7]. Once HA has been terminated, such adaptations gradually disappear [8] and whilst much is known about whole-body sweat secretion, there is limited information about LSR and the content of sweat during HA and the decay period. If an individual is re-exposed to a heat stimulus via heat re-acclimation (HRA) following a decay period, there is some evidence to suggest a faster accrual of the lost adaptations [9,10]. However, this information is limited to core temperature, heart rate and whole-body sweat loss (WBSL). LSR and the content of sweat during HA, the decay period and HRA are poorly understood.

Following HA, WBSL adaptations have been reported to be completely lost after a 26- [9] or 28-day decay period [11]. After such a decay period, an equal HRA exercise protocol to HA resulted in similar or better WBSL adaptations but within a shorter time frame [9,12]. With repeated active heat exposure, it has been suggested that this occurs via a molecular HA memory [13]. The HA memory is considered to be a reversible regulatory system that is activated by common signaling molecules and in which a second exposure (i.e., HRA) elicits a faster response. It is plausible that the regulatory HA memory system is also activated by passive heat exposure [14]. However, whether similar local sudomotor adaptations occur during active and passive HRA remains unknown. Previous research observed a higher LSR during a single active compared to passive heat exposure. As from a physiological control perspective core temperature is the main driver of sweat production [15], this finding was attributed to a higher core temperature in active heat exposure [16]. Furthermore, Gerrett et al. [17] observed higher concentrations of the water regulatory hormone aldosterone in saliva after active compared to passive heat exposure. Due to the suggested positive relation between aldosterone concentration and the eccrine sweat glands’ reabsorption rate of ions [17], it could be expected that active HRA induces higher sodium and chloride reabsorption rates. These higher reabsorption rates would result in lower sweat sodium and chloride concentrations in active compared to passive HRA. Unlike sodium and chloride, potassium is probably not reabsorbed from precursor sweat in the distal duct of the gland [18]. As a result, sweat potassium concentrations appear to be relatively constant despite changing the environmental conditions or exercise intensity [19]. Therefore, an active or passive HRA protocol will most likely not affect sweat potassium concentrations either.

Another sweat component that has recently gained interest is lactate. Engineers and developers have integrated sweat lactate detection systems in wearable sensors to monitor health and performance [20]. However, sweat lactate appears to be a by-product of the sweat glands glycolytic ATP re-synthesis [21], baring no established relation with health or performance. Yet the presence of lactate in sweat is interesting and may help to provide important insight into the regulatory mechanisms associated with the formation of sweat. The sweat glands’ energy dependent processes are the formation of precursor sweat in the sweat glands’ coil and reabsorption of sodium in the duct [22,23]. The repeated heat stimulus with HA and HRA is thought to result in higher secretory activity and higher sodium reabsorption rates [7]. Since these processes are energy driven, an increased sweat lactate concentration could be observed. On the other hand, repeated heat exposure generally allows for more efficient functioning [24]. In terms of sweat gland metabolism, efficiency could be reflected in emitting greater volumes of sweat relative to ATP production, secreting less lactate in the presence of a higher LSR.

The purpose of the current study was to investigate LSR and sweat sodium, chloride, lactate and potassium concentrations before and after HRA using active or passive protocols and comparing this to a control condition (no HRA). It was hypothesized that 1) active HRA would result in greater sudomotor adaptations, such as a higher LSR and lower sweat sodium, chloride and lactate concentrations in sweat than passive HRA and 2) that potassium concentrations would remain unchanged following either active or passive HRA. The outcomes of the present study could help to improve our understanding of the underlying mechanisms of sudomotor adaptations and can potentially be used in setting up practical recommendations for athletes regarding the most convenient adaptation strategy to the heat.

## Materials and methods

### Ethical approval

Full ethical approval was granted by the Ethics Committee of the Faculty of Behavioural and Movement Sciences of the Vrije Universiteit Amsterdam (VCWE-2018-160R1). The study was conducted in accordance with the guidelines of the revised *Declaration of Helsinki* (2013). Participants were informed about all procedures and potential risks were identified before written informed consent was obtained.

### Participants

Fifteen non-acclimatized participants visited the laboratory for the same controlled hyperthermia (CH) HA protocol, followed by a 28-day decay period. Thereafter, participants were divided over three HRA groups: six of them were re-exposed to CH (CH-CH), five to hot water immersion (CH-HWI) and four were control (CH-CON); Table 1). Based on their peak oxygen uptake (VO_2peak_), peak power output and training frequency, participants were either classified as recreationally trained or trained (Table 1) [25,26]. No significant differences were observed between participant characteristics (Table 1). Participants had not encountered hot air temperatures (> 25°C) for > 3 months, were nonsmokers, had no history of heat-related illnesses, cardiovascular complications and did not have any known issues with thermoregulation. Only the female participant in CH-CON was taking medication (70 mg alendronic acid weekly, 500 mg calci-chew and 7.5 mg mirtazapine daily) throughout the study. Female menstrual cycle phase was recorded but not controlled for as this would have been unfeasible given the required timing of each part of the protocol (10-day HA, 28-day decay and 5-day HRA consecutively). Furthermore, participants were instructed to refrain from alcohol and strenuous exercise 24 h prior to the experiments, to avoid excessive caffeine consumption during the entire protocol, and to refrain from caffeine and fast 2 h prior to testing. To promote hydration, participants were asked to consume 500 mL of water the night before the experiments and another 10 mL·kg^−1^ body mass of water in the 3 h prior to the experiment. Participants replicated their food and fluid intake starting 24 h before each HST.

**Table 1.** Mean (SD) participants characteristics of the three groups. No differences were observed between the heat re-acclimation strategies for any variables (P > 0.05)

	CH-CON (n = 4, 3 M, 1 F)	CH-CH (n = 6, 4 M, 2 F)	CH-HWI (n = 5, 3 M, 2 F)
Age (yr)	34 ± 9	37 ± 5	29 ± 8
Height (cm)	184.7 ± 3.5	179.3 ± 3.6	183.4 ± 9.1
Weight (kg)	78.9 ± 3.1	78.3 ± 6.9	75.0 ± 10.9
BSA (m^2^)	2.02 ± 0.1	1.97 ± 0.1	1.97 ± 0.2
VO_2peak_ (mL⋅kg^−1^⋅min^−1^)	50.6 ± 7.9	49.7 ± 8.9	49.7 ± 9.5
Training status	4RT, 2 T	2RT, 2 T	3RT, 2 T

M, Males; F, Females; BSA, Body surface area; CH-CON, control group; CH-CH, Heat re-acclimation with controlled hyperthermia; CH-HWI, Heat re-acclimation with hot water immersion; RT, Recreationally trained; T, Trained [25,26].

### Experimental design

**Figure 1.**
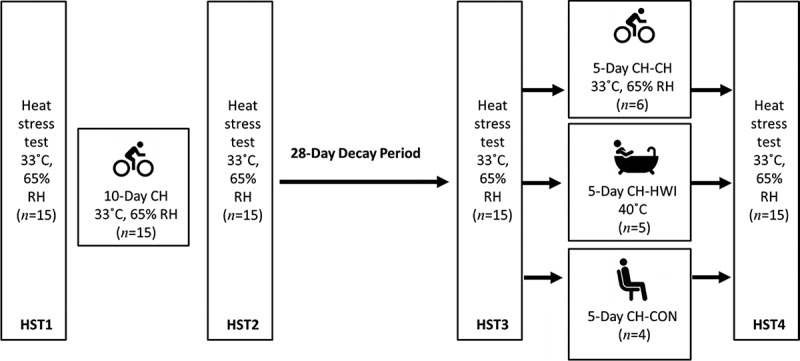
Schematic overview of the study. HST: heat stress test, CH: controlled hyperthermia, CH-CH: heat re-acclimation with controlled hyperthermia, CH-HWI: heat re-acclimation with hot water immersion, CH-CON: heat re-acclimation control group (no heat re-acclimation), RH: relative humidity. Sweat samples were taken during HST1, 2, 3 and 4

All experiments were performed during Northern Hemisphere winter time (Jan-Apr 2019) to limit acclimatization through exposure to high environmental temperatures. HST, HA and active HRA (CH-CH) were completed in a climate chamber (b-Cat, Tiel, The Netherlands), set at 33°C and 65% relative humidity, and at the same time of day (± 3 h) per participant to control for circadian rhythms. The research design is presented in Figure 1, with participants visiting the laboratory 19 times. During each HST, participants cycled (Lode Excalibur, Groningen, The Netherlands; or Wattbike Pro, Duivendrecht, The Netherlands) at 1.5 W·kg^−1^ body mass for 35 min [27]. Thereupon, they had a 5-min break in which they drunk 3 mL·kg^−1^ body mass of water and subsequently, performed a graded exercise test until volitional exhaustion. Starting power output of the graded exercise test was 1.5 W·kg^−1^ body mass, thereafter increasing with 25 W·min^−1^ [28]. During HA participants cycled at an intensity chosen such that a T_re_ of 38.5°C was reached within approximately 40 min (referred to as “thermal drive”). As participants adapted during the HA and HRA (CH-CH only) days, exercise load was increased so that T_re_ still reached 38.5°C within ~40 min. After that, T_re_ was kept slightly above 38.5°C for 60 min (referred to as “thermal maintenance”; i.e., CH) [29]. This was accomplished by the researcher adapting the exercise load and resting where necessary. Water consumption was allowed *ad libitum* and the volume consumed was recorded. In the present study, HA and active HRA were induced by a CH protocol, as this has been observed to elicit equal physiological adaptations compared to a fixed workload protocol but requires lower exercise duration and intensity [30]. During the 28-day decay period, participants performed their regular exercise activities, but were not allowed to have heat exposure. Decay was followed by five consecutive days of HRA. Passive HRA consisted of a hot water immersion protocol, chosen based on the practical feasibility for athletes to implement. For the hot water immersion protocol, a water temperature of ~40°C for 40 min was utilized; participants were submerged to the neck for 10 min in ~40°C water, followed by submersion to the waist for 2 min by sitting on a chair in the bath. Thereafter, 8 min of submersion to the neck and 2 min to the waist were alternated until a total time of 40 min. When exiting the bath, participants rested in supine position for 5 min, or longer if needed, to prevent dizziness.

### Measurements

Upon arrival to the laboratory, participants provided a urine sample. Urine specific gravity (USG) was measured with a handheld refractometer (Atago PAL-S, Bellevue, USA) to make sure participants were hydrated (USG ≤ 1.025) [31]. Despite the hydration instructions, six participants did not meet the criteria for hydration before one of the HST and had to drink 5 mL·kg^−1^ body mass before resuming the experiment. Prior to each heat exposure, a rectal temperature (T_re_) probe (MSR, Seuzach, Switzerland; or Yellow Springs Instruments, Ohio, USA) was self-inserted 10 cm past the anal sphincter. Except from the hot water immersion, skin temperature (T_sk_) (iButton DS1922, Maxim Integrated Products, California, USA) was measured from the chest, forearm, thigh and calf, and a weighted mean T_sk_ was calculated [32].

Upper arm and upper back sweat (covering zones 19 and 13 described by Gerrett et al. [33]) were collected during the first and second 15 min of cycling at 1.5 W·kg^−1^ body mass and during the first 15 min of the graded exercise test (whenever possible; otherwise until exhaustion) of HST1–4 using the absorbent patch technique [34]. These skin sites were chosen based on the reported high LSR on the back [35], which most likely allowed for collection of enough sweat for chemical analysis. Furthermore, previous studies have most often measured LSR and sweat content from these sites, allowing us to compare our results with previous findings. The arm is also a preferred site for the application of wearable sweat monitoring devices.

We aimed for three different LSR by sequentially collecting the three sweat samples (from now on referred to as “sample order”). The sample duration of 15 min was chosen to collect enough sweat for chemical analysis but to prevent saturation of the patches. To prevent hidromeosis underneath the patches, they were visually inspected and removed earlier if necessary. This is a modified version of the absorbent patch technique introduced by Smith and Havenith [35], that was later adopted by Baker and colleagues [36], since in the present study not only LSR was determined but also enough sweat for chemical analysis had to be collected. Before application, the skin was cleaned with alcohol, deionized water and dried with gauze pads. The researcher wore gloves when applying and removing the patches and removed the absorbent material with cleaned tweezers. After sweat collection, the absorbent material (Cutisoft, BSN Medical, Almere, The Netherlands) was placed in a sealed tube (Salivette, Sarstedt, Nümbrecht, Germany) using tweezers, centrifuged at 1800 *g* for 5 min and frozen at −20°C until analysis. Patches were carefully replaced at the same location by using marks on the skin. The absorbent material was covered with an impermeable fabric (Parafilm M, Bemis, Saint Louis, USA) and fixed to the skin using a porous adhesive on top of that (Fixomull stretch, BSN Medical, Almere, The Netherlands). Concentrations of sodium, chloride and potassium were determined by ion-selective electrodes (Cobas 8000 modular analyzer, Roche, Almere, The Netherlands). Lactate concentration was analyzed enzymatically (Cobas LACT2, Roche, Almere, The Netherlands). Limits of detection, coefficients of variation and sample volumes of the analyses are shown in. Background sodium, chloride, lactate and potassium in the patches was determined and concentrations were corrected accordingly (sodium −7.9 mmol·L^−1^, chloride −6.6 mmol·L^−1^, no corrections needed for lactate and potassium). LSR was calculated according to:

$$LSR=\left(\left(m_{wet}-m_{dry}\right)/t\right)/SA$$

Where $m_{wet}$ refers to mass of the wet patch (mg) (Analytical balance 1419MP8-1, Sartorius, Goettingen, Germany, accuracy: 1 mg), $m_{dry}$ to its dry mass (mg), SA to surface area of the covered skin (cm^2^) and t to application time (min). LSR is presented in mg·cm^−2^·min^−1^. WBSL for each day of the CH-CH and CH-HWI protocols was calculated by correcting the difference between pre- and post-nude body mass for drinking volume (SATEX 34 SA-1 250, Weegtechniek Holland, Zeewolde, The Netherlands). The corresponding WBSR (in L·h^−1^) was calculated by correcting WBSL for heat exposure time.

### Data analysis

To assess whether general participant characteristics differed between HRA groups, a one-way ANOVA was used with group (CH-CH, CH-HWI and CH-CON) as factor. Generalized Estimating Equations (GEE) modeling [37] was used to evaluate LSR and sweat sodium, chloride, lactate and potassium concentration over each phase (HST1–4) using IBM SPSS Statistics 26.0. This approach to regression analysis considers measurements within participants (HST1–4 and sample order one–three) as repeated measures and accounts for the dependency of these values. GEE does not assume normally distributed data and accommodates for missing data points. An exchangeable correlation structure was used. The analysis was carried out for the arm and back separately because of previous reported large regional differences in LSR and sweat composition [38]. The independent variables were phase (HST1–4), sample order (one–three) and HRA group (CH-CH, CH-HWI, CH-CON). The dependent variables were LSR, sweat sodium, chloride, lactate and potassium concentration. Interactions of phase *x* sample order and LSR, sweat sodium, chloride, lactate and potassium concentration were included in the analysis of the model when significant (*P* < 0.05). HST2 and HST1 were compared to assess whether HA was successful, HST3 and HST2 to confirm decay, HST3 and HST1 to assess whether decay was complete and finally, HST4 and HST3 to assess whether HRA was successful. Following the main analysis, that included all phases, a separate GEE including HST3 and HST4 was used for specific HRA comparisons between groups. The thermal impulse for T_re_ on each HRA day for the CH-CH and CH-HWI group was calculated according to:

[39] $$Thermal\;impulse\;for\;T_{re}=\;\int \left(T_{re-i}-T_{re-0}\right)dt\left[{}^{\circ}C\cdot min\right]$$

Where T*_re-i_* is T_re_ at time point i (°C), T*_re-0_* represents baseline T_re_ (°C) and *dt* is the duration of the stimulus (min). GEE modeling was again used to evaluate the effect of HRA group on WBSL and WBSR. The independent variables were HRA day (one–five) and HRA group (CH-CH or CH-HWI). The dependent variables were WBSL and WBSR.

## Results

All fifteen participants completed the entire protocol. Sweat was successfully collected during HST1, 2, 3 and 4 (pre-HA, post-HA, pre-HRA and post-HRA).

### LSR, sweat sodium, chloride, lactate and potassium

There were significant main effects of sample order on LSR, sweat sodium, chloride and lactate concentration on the arm and back (*P* < 0.05). However, interactions of phase *x* sample order on LSR, sweat sodium, chloride, lactate and potassium were non-significant on the arm and back (*P* ≥ 0.071). This indicates that LSR and concentrations of components were different, but that patterns over phases of the protocol (HST1–4) were similar for the three different samples. Therefore, sample order data (three samples during each heat exposure) is pooled in further analysis.

There were significant main effects of phase on LSR, sweat sodium, chloride and lactate concentrations on the arm and back (*P* < 0.001; Figures 2, Figures 3). LSR on the arm was significantly higher in HST4 than HST3 (*P* = 0.014). For LSR measured on the back, HST2 was significantly higher than HST1 (*P* < 0.001), and HST4 than HST3 (*P* < 0.001), indicating that HA and HRA were successful. HST3 was significantly lower than HST2 (*P* < 0.001), but not compared to HST1 (*P ≥ *0.078), indicating that decay was complete (Figures 2, Figures 3). For sweat sodium and chloride on the arm and back and lactate on the arm, concentrations in HST2 were significantly lower than in HST1 (*P* < 0.001), HST4 than HST3 (*P* < 0.001), and HST3 was significantly higher than HST2 (*P* < 0.001), but not compared to HST1 (*P* ≥ 0.051). For sweat potassium, no significant main effects of phase were found on the arm and back (*P* = 0.050, *P* = 0.056; Figures 2, Figures 3).

**Figure 2.**
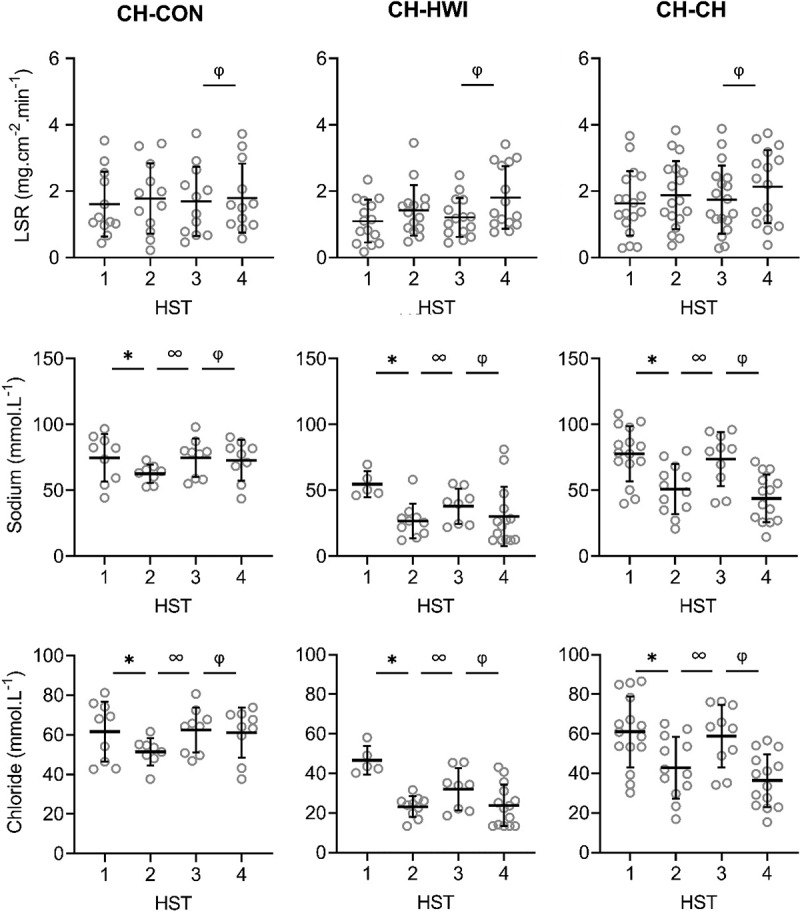
Local sweat rate (LSR), sweat sodium, chloride, lactate and potassium concentrations on the upper arm during heat stress test (HST) 1–4. HST1 was performed pre-heat acclimation (HA), HST2 post-HA, HST3 pre-heat re-acclimation (HRA) (after a 28-day decay) and HST4 post-HRA. Data is shown separately for control (CH-CON; *n* = 4), HRA by hot water immersion (CH-HWI; *n* = 5) and HRA by controlled hyperthermia (CH-CH; *n* = 6). Data includes three samples per participants during each HST. Grey circles represent individual data, whilst solid black lines represent means and standard deviations. * indicates main effects (*P* < 0.05) of phase between HST1 and HST2. ∞ indicates main effects of phase between HST2 and HST3. φ indicates main effects of phase between HST3 and HST4

**Figure 2.**
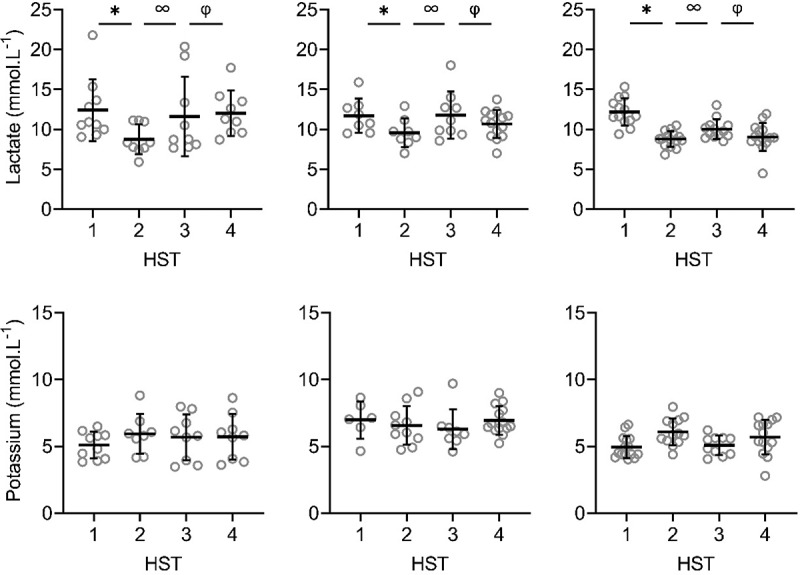
(Continued)

**Figure 3.**
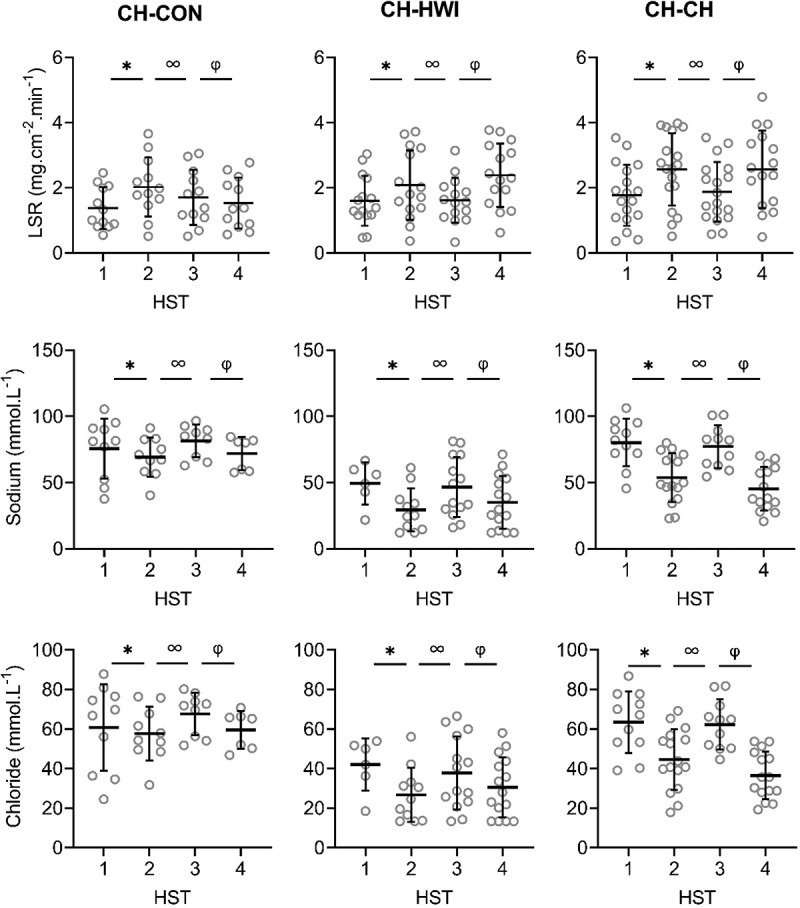
Local sweat rate (LSR), sweat sodium, chloride, lactate and potassium concentrations on the upper back during heat stress test (HST) 1–4. HST1 was performed pre-heat acclimation (HA), HST2 post-HA, HST3 pre-heat re-acclimation (HRA) (after a 28-day decay) and HST4 post-HRA. Data is shown separately for control (CH-CON; *n* = 4), HRA by hot water immersion (CH-HWI; *n* = 5) and HRA by controlled hyperthermia (CH-CH; *n* = 6). Data includes three samples per participants during each HST. Grey circles represent individual data, whilst solid black lines represents mean and standard deviations. * indicates main effects (*P* < 0.05) of phase between HST1 and HST2. ∞ indicates main effects of phase between HST2 and HST3. φ indicates main effects of phase between HST3 and HST4

**Figure 3.**
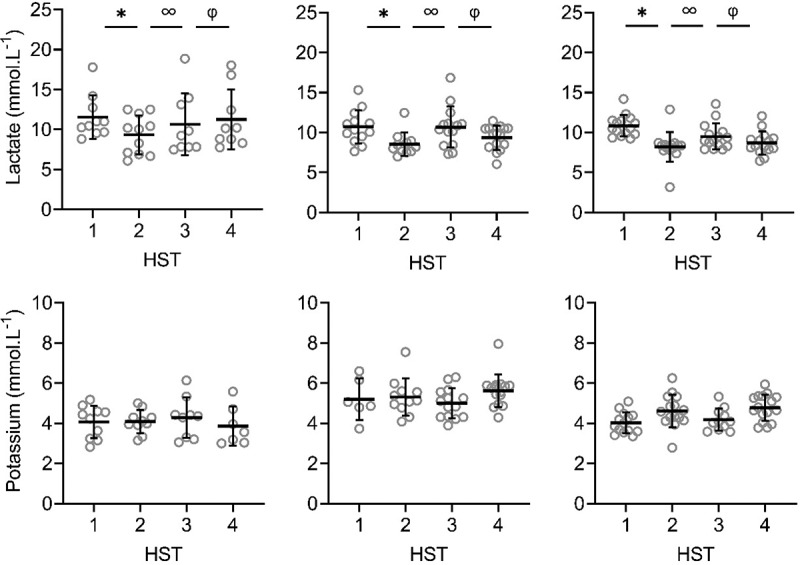
(Continued)

There were significant main effects of HRA group on sweat sodium, chloride and lactate concentration on the arm and back (*P* ≤ 0.029), but not for LSR (*P* ≥ 0.519). Interactions of phase *x* HRA group on LSR, sweat sodium and chloride were significant on the arm and back (*P* ≤ 0.003). Post-hoc testing revealed that from HST3 to HST4, the LSR increases were larger for both CH-CH (arm 1.7 ± 1.0 to 2.1 ± 1.1 mg.cm^−2^.min^−1^; back 1.9 ± 0.9 to 2.6 ± 1.2 mg.cm^−2^.min^−1^) and CH-HWI (arm 1.2 ± 0.6 to 1.8 ± 0.9 mg.cm^−2^.min^−1^; back 1.6 ± 0.7 to 2.4 ± 0.9 mg.cm^−2^.min^−1^) than CH-CON (arm 1.7 ± 1.0 to 1.8 ± 1.0 mg.cm^−2^.min^−1^; back 1.7 ± 0.8 to 1.5 ± 0.7 mg.cm^−2^.min^−1^, *P ≤ *0.010; Figures 2, Figures 3), but CH-CH and CH-HWI were not different (*P* ≥ 0.148). The decreases in sodium and chloride were larger in CH-CH (arm 73.8 ± 19.4 to 43.9 ± 17.4 mmol.L^−1^; and 58.9 ± 15.0 to 36.4 ± 12.9 mmol.L^−1^; back 77.3 ± 15.4 to 45.4 ± 15.8 mmol.L^−1^; and 62.3 ± 12.1 to 36.5 ± 11.6 mmol.L^−1^) compared to CH-HWI (arm 38.0 ± 12.5 to 30.2 ± 21.7 mmol.L^−1^; and 32.0 ± 9.9 to 23.8 ± 10.1 mmol.L^−1^; back 46.7 ± 21.9 to 35.0 ± 19.2 mmol.L^−1^; and 37.9 ± 17.7 to 30.6 ± 14.7 mmol.L^−1^) and CH-CON (arm 74.8 ± 13.7 to 72.9 14.6 mmol.L^−1^; and 62.4 ± 10.6 to 61.1 ± 11.9 mmol.L^−1^; back 81.4 ± 11.6 to 72.0 ± 15.8 mmol.L^−1^; and 67.6 ± 10.1 to 59.6 ± 8.8 mmol.L^−1^, *P* ≤ 0.001; Figures 2, Figures 3). The decreases in CH-HWI and CH-CON were not different from each other (*P* ≥ 0.265). The interaction of phase *x* HRA group on lactate and potassium were only significant on the back (*P* < 0.001). The decreases in upper back sweat lactate concentrations from HST3 to HST4 were significantly larger in CH-CH (9.5 ± 1.6 to 8.7 ± 1.4 mmol.L^−1^) and CH-HWI (10.7 ± 2.5 to 9.4 ± 1.5 mmol.L^−1^), compared to the increase in CH-CON (10.7 ± 2.5 to 11.3 ± 3.6 mmol.L^−^1, *P* < 0.001; Figures 2, Figures 3). CH-CH and CH-HWI were not different from each other (*P* = 0.284). Post-hoc testing also revealed that both CH-CH (4.2 ± 0.5 to 4.7 ± 0.6 mmol.L^−1^) and CH-HWI (5.0 ± 0.7 to 5.6 ± 0.8 mmol.L^−1^) showed larger potassium increases on the back than the decreases in CH-CON (4.3 ± 1.0 to 3.9 ± 0.9 mmol.L^−1^, *P* ≤ 0.002) following HRA (Figures 2, Figures 3), but CH-CH and CH-HWI were not different (*P* = 0.967)

### T_re_, thermal impulse, WBSL, WBSR

There were significant main effects of HRA group (CH-CH and CH-HWI) on T_re_, thermal impulse and WBSL (*P ≤ *0.022), but not of HRA group on WBSR (*P* = 0.560) (Table 3). T_re_, thermal impulse and WBSL were higher in CH-CH compared to CH-HWI (*P* < 0.001). In CH-CH, mean T_sk_ was ~36°C (Table 3), whilst T_sk_ was not measured in CH-HWI. However, water temperature was ~40°C so we assumed mean T_sk_ to be higher in CH-HWI compared to CH-CH.Table 2

**Table 2.** Characteristics of the analysis for sweat sodium, chloride, lactate and potassium

	LOD (mmol·L^−1^)	CV (%)	Sample volume (μL)
Sodium	0.4	<1	15
Chloride	0.4	<2.2	15
Lactate	0.2	<3.6	2
Potassium	0.2	1.0–1.3	15

LOD, limit of detection; CV, coefficient of variation.

**Table 3.** Mean and standard deviation of physiological parameters per day of heat re-acclimation by controlled hyperthermia (results are shown for thermal maintenance) and hot water immersion. Skin temperature was not measured during hot water immersion. * denote significant (*P* < 0.05) differences from controlled hyperthermia

		HRA1	HRA2	HRA3	HRA4	HRA5
T_re_ (°C)	CH-CH	38.67 (0.07)	38.55 (0.04)	38.66 (0.06)	38.62 (0.04)	38.61 (0.05)
	CH-HWI	38.16 (0.40)	38.17 (0.48)	38.08 (0.52)	38.21 (0.54)	38.18 (0.58)
Thermalimpulse (°C·min)	CH-CH	3530 (284)	3542 (262)	3504 (265)	3529 (355)	3580 (321)
	CH-HWI	1329 (15)*	1330 (7)*	1319 (20)*	1331 (8)*	1331 (9)*
Mean T_sk_ (°C)	CH-CH	36.2 (0.3)	36.2 (0.2)	36.0 (0.3)	35.9 (0.3)	36.0 (0.2)
	CH-HWI	-	-	-	-	-
WBSL (L)	CH-CH	2.3 (0.8)	2.4 (0.8)	2.5 (0.7)	2.5 (1.0)	2.7 (0.9)
	CH-HWI	1.0 (0.5)*	1.1 (0.6)*	1.2 (0.6)*	1.3 (0.6)*	1.3 (0.6)*
WBSR (L·h^−1^)	CH-CH	1.4 (0.4)	1.5 (0.4)	1.5 (0.4)	1.5 (0.5)	1.6 (0.5)
	CH-HWI	1.5 (0.7)	1.7 (1.0)	1.7 (1.0)	1.9 (0.9)	1.9 (1.0)

T_re_, rectal temperature; Mean T_sk_, mean skin temperature; WBSL, whole-body sweat loss; WBSR, whole-body sweat rate; HRA, heat re-acclimation; CH-CH, heat re-acclimation by controlled hyperthermia; CH-HWI, heat re-acclimation by hot water immersion

## Discussion

This is, to our knowledge, the first study to assess LSR and sweat sodium, chloride, lactate and potassium concentrations before and after an active or passive HRA protocol. Increases in LSR on the arm and back, decreases in sweat lactate on the back and increases in potassium concentrations on the back were similar in CH-CH and CH-HWI but larger than in CH-CON (Figures 2, Figures 3). Decreases in sweat sodium and chloride concentrations were larger in CH-CH than CH-HWI and CH-CON (whilst the  latter two did not differ from each other) on both measurement locations. Thus, our hypothesis that active HRA by controlled hyperthermia (CH-CH) induces greater local sudomotor adaptations compared to passive HRA by hot water immersion (CH-HWI) was accepted for sodium and chloride, but rejected for LSR, lactate and potassium.

### Induction of HA and HRA

HA successfully induced higher LSR on the back and lower sweat sodium, chloride and lactate concentrations on the arm and back, whilst sweat potassium was not affected by the HA protocol on both measurement locations (Figures 2, Figures 3). During the 28-day decay period without heat exposure, LSR decreased on the back, whilst sweat sodium, chloride and lactate increased for all three groups on both measurement locations (Figures 2, Figures 3). For sweat sodium, chloride and lactate on both skin sites, decay was complete following 28-days without heat exposure, as indicated by a non-significant difference between HST3 and HST1. As there was no adaptation in sweat potassium following HA in the first place, there was no decay either. HRA successfully re-induced higher LSR and lower sweat sodium, chloride and lactate concentrations on both measurement locations Like with HA, sweat potassium was not affected following HRA on both measurement locations (Figures 2, Figures 3).

Following HA, the LSR increase on the arm was not statistically significant but potentially is physiologically important. A 0.25 mg·cm^−2^·min^−1^ or ~18% increase in LSR equals ~0.3 L of sweat and ~728 kJ of heat loss from a body surface area of 2 m^2^ during 55 min of cycling in the heat. For comparison, the statistically significant LSR increase on the back was 0.64 mg·cm^−2^·min^−1^ (~41%). Since sudomotor adaptations usually take the longest time to acquire [24], perhaps more time was required for the adaptations to be statistically significant, especially in areas of lower sweat production such as the extremities [38]. The absence of a significant LSR elevation on the arm is in contrast to previous research suggesting LSR redistribution to the periphery following HA [39–42]. It could be that the compensability of our HST affected the LSR outcomes. Per the heat balance theory, our HST were likely considered compensable. In relation to this, Jay et al. [41] found that compensable heat stress elicited smaller increases in LSR on the arm and back when compared to uncompensable heat stress following HA, which could potentially explain the absence of a significant LSR elevation in the present study.

Like in the present study, previous research reported lower sweat sodium and chloride concentrations in the presence of an elevated LSR following HA [5–7]. The potential underlying mechanism is an improved reabsorption of sodium and chloride in the sweat glands, ducts through epithelial sodium channels [43,44] and cystic fibrosis transmembrane conductance regulators [43], respectively. The reduced sweat lactate concentrations may relate to the significantly higher LSR as sweat lactate and LSR are inversely proportional due to dilution [22]. HA and HRA may also have provided an adequate stimulus for emitting greater volumes of sweat relative to ATP production, indicating improved sweat gland efficiency [21,23] or greater reliance on the aerobic metabolic pathways [23]. We cannot confirm or contradict these theories here but they certainly raise interesting avenues for future research. The absence of a phase effect on potassium is in line with our hypothesis and possibly due to the lack of a reabsorption mechanism for potassium in the sweat glands’ ducts, causing relatively constant levels in sweat [19].

### Sudomotor adaptations to active versus passive HRA

In contrast to our hypothesis, LSR increases were not different following active or passive HRA as measured during a standardized HST before and after the HRA protocols (Figures 2, Figures 3). Previous research suggested that T_re_ is the main driver of sweating and T_sk_ only has a modifying effect [15]. Considering the chronically higher T_re_ in CH-CH (Table 3), larger LSR increases were expected. Secondly, previous research reported that post-exercise LSR was increased on non-glabrous skin via metaboreflex stimulation [45]. The involved muscle metaboreceptors respond to an increase in the muscles metabolic products and stimulate an increase in sudomotor activity. The latter mechanism only applies to the active CH-CH group in this study. The effect of a chronically higher T_re_ and the metaboreflex stimulation on sweat gland activity would suggest a higher LSR in CH-CH, which was not observed here (Figures 2, Figures 3). On the other hand, there is direct evidence of peripheral modifications after HA. For example, cholinergic sensitivity of the glands and cutaneous microvasculature were improved [46,47]. Whether these changes were driven by an elevated T_sk,_ T_re_, or a combination remains unclear. Earlier work by Regan et al. [48] highlighted the need for an external heat stress (i.e., elevated T_sk_) in addition to an elevated core temperature during HA to fully adapt. It may be that in the present study, despite the lower T_re_ in CH-HWI, the higher mean T_sk_ provided the additional peripheral stimuli to induce sudomotor adaptations with a similar outcome to CH-CH.

From a physiological control perspective, the similar LSR following active or passive HRA may therefore be explained by the predominance of central thermoregulatory adaptations in CH-CH, induced by a relatively high T_re_ and thermal impulse [15], and a predominance of peripheral modifications to the thermo-effectors in CH-HWI [46,47] , induced by a relatively high mean T_sk_ (Table 3). From a biophysical control perspective, required evaporation determines sweat secretion rate independent of the absolute T_re_ and T_sk_ [49]. Due to the high metabolic heat production in active HRA, required evaporation is high as well, whilst required evaporation in passive HRA is high because dry heat loss is impeded but there is dry heat gain. These potentially balance each other out in the present study. In line with our findings and the theories above, Kondo et al. [16] observed a similar sweat gland output during a single active and passive heat exposure in the presence of a higher core temperature and lower mean T_sk_ in active heat exposure.

Both WBSL and WBSR were measured and reported to provide insight in the whole-body sudomotor adaptations that occurred during five consecutive HRA days. WBSR was found to be similar between CH-CH and CH-HWI, but WBSL was higher in CH-CH compared to CH-HWI (Table 3). This can be explained by the twofold longer heat exposure time for the CH-CH group, which is also reflected by the thermal impulse. First and foremost, sweat glands must be active to adapt (i.e., be able to secrete more sweat) during HA [50]. These sudomotor adaptations may be elicited above a certain sweat rate, rather than total sweat volume. Support of this idea comes from work by Taylor et al. [51], suggesting that a WBSR of 0.4–0.8 L·h^−1^ is required to gain sudomotor adaptations. In the present study, WBSR exceeded this suggested threshold in both CH-CH and CH-HWI (Table 3), which potentially contributes to the explanation of similar sudomotor adaptations following active or passive HRA. With a comparable WBSR after active or passive HRA, similar amounts of heat can in theory be removed from the body. This has implications in sports or occupational settings where prolonged periods of heat exposure occur frequently.

Regarding sweat composition, we observed a larger conservation response for sweat sodium and chloride in CH-CH compared to CH-HWI and CH-CON on both the arm and back. Previous research observed higher maximal ion reabsorption rates in the eccrine sweat glands during a single active compared to passive heat exposure [17]. These higher maximal reabsorption rates were likely related to higher concentrations of the water regulatory hormone aldosterone during active (exercise intensity >50% VO_2max_) compared to passive heat exposure [17]. When it comes to repeated heat exposure, Buono et al. [7] reported adaptations in the sweat glands, reabsorption rate following active HA by CH. These findings indeed suggest that in the present study, active CH-CH would lead to greater adaptations (i.e., reabsorption rates) in sweat sodium and chloride compared to passive CH-HWI, causing lower levels of sodium and chloride in sweat at the skin surface. Here, the relatively high mean T_sk_ in the CH-HWI group may not have been sufficient to induce additional sudomotor adaptations to an elevated T_re_, with a similar sudomotor outcome to CH-CH. In relation to this, Gerrett et al. [17] observed a T_sk_ dependency of the eccrine sweat glands’ maximal reabsorption rate: with a 6°C T_sk_ elevation, higher maximal reabsorption rates were observed, whereas increasing T_sk_ by 3°C did not elucidate a difference in maximal reabsorption rate. As in the present study water temperature was ~40°C and mean T_sk_ in CH-CH was ~36°C (Table 3), a ~ 4°C mean T_sk_ difference between active and passive HRA could have been present. Since a 6°C T_sk_ difference was the minimum to show a significant higher reabsorption rate, the ~4°C in the present study may not have been sufficient. Assuming that reabsorption rates respond similarly to repeated heat exposure, the differences in sweat sodium and chloride between CH-CH and CH-HWI are potentially caused by larger adaptations in reabsorption rates induced by water regulatory hormones in CH-CH.

Sweat lactate concentrations decreased similarly in CH-CH and CH-HWI, contradicting our hypothesis that active HRA (CH-CH) would induce lower sweat lactate concentrations. This was, however, hypothesized to relate to a higher secretory activity in CH-CH, which was not observed either: WBSR and LSR were similar between conditions (Table 3; Figures 2, Figures 3). Assuming that the sweat glands’ metabolism was similar in both conditions due to the similar secretory activity, lactate concentrations were as well. Lastly, adaptations in sweat potassium concentrations on the back were larger in CH-CH and CH-HWI than CH-CON. In addition, the concentrations in CH-CH and CH-HWI increased whilst in CH-CON they decreased. This corresponds to the LSR patterns on the back, suggesting that a higher LSR causes accumulation of potassium in the absorbent patches.

### Limitations

It would have been interesting to implement a CH protocol for passive HRA as well. In this way, T_re_ would have been similar in both HRA protocols and the adaptation mechanisms could be better elucidated. However, passive CH is a physically challenging protocol to sustain and for the current study the hot water immersion protocol was chosen based on practical feasibility for athletes. Future research may consider comparing active and passive HRA when core temperature is matched.

The sample size of our HRA groups was small (*n* = 4–6) which inflates the risk of type M errors [52] and whilst statistical analysis has been conducted, the readers should view the data as preliminary findings.

HRA groups also included an unbalanced mix of males and females. It is known that core temperature is regulated ~0.4°C higher in the luteal phase of the menstrual cycle and that simultaneously the core temperature threshold for sweating and vasodilation is increased [53]. As we did not control for menstrual cycle phase or use of oral contraceptives, sex could have had an effect on our LSR results. Sweat composition is most likely unaffected by menstrual cycle phase [54]. Future research should preferably include more participants of both sexes.

Another potential limitation could be that we continuously measured LSR by using absorbent patches. Smith & Havenith [35] first established this technique and sampled at the end of exercise for 5 min to measure sweat volume. However, in order to collect sufficient sweat to expel sweat from the absorbent patch for chemical analysis, such short sampling times are not feasible. By covering the skin for prolonged periods of time (3 *x* 15 min in the present study) increases in local T_sk_ are unavoidable. However, previous research concluded that variations in T_sk_ do not predict variations in LSR [35,55], which may also apply to the present study. Support for this argument comes from research by Nadel et al. [15,56], who concluded that T_sk_ modifies sweating whilst core temperature is considered the main driver of sweating. Since our patches were small (25 cm^2^), no effect of its application on core temperature is expected.

Even though there were no between-group differences in general participant characteristics, baseline LSR and sweat composition data varied considerably both between and within groups. It is plausible to assume that a higher LSR and an already diluted sweat solution allow for less adaptation. However, we are not aware of any study supporting this theory. LSR and sweat composition are affected by many factors [3,4] and a lot of unknowns exist on how final sweat composition arises. Future research should investigate the effect of baseline LSR and sweat composition on the sudomotor adaptations responses.

### Conclusions

Active heat re-acclimation by controlled hyperthermia and passive heat re-acclimation by hot water immersion resulted in similar increases in local sweat rate on the arm and back as measured during a standardized heat stress test before and after heat re-acclimation. Such findings may be explained by the predominance of central thermoregulatory adaptations in active heating and a predominance of peripheral modifications to the thermo-effectors in passive heating. Secondly, active heat re-acclimation led to more conservation of sodium and chloride than passive heat re-acclimation (similar to no heat re-acclimation) on both the arm and back, which may be caused by the effects of exercise on reabsorption rates of the eccrine sweat glands. The decreases in sweat lactate and increases in potassium on the back were larger in active compared to passive heating, potentially due to dilution and accumulation, respectively.
